# Clinical application of magnetic anchor technique in laparoscopic cholecystectomy: the first retrospective study in China

**DOI:** 10.3389/fsurg.2023.1335805

**Published:** 2024-01-05

**Authors:** Boyan Tian, Miaomiao Zhang, Yuxiang Ren, Yuhan Zhang, Yi Lyu, Xiaopeng Yan

**Affiliations:** ^1^ Department of Hepatobiliary Surgery, The First Affiliated Hospital of Xi’an Jiaotong University, Xi’an, China; ^2^ National Local Joint Engineering Research Center for Precision Surgery & Regenerative Medicine, The First Affiliated Hospital of Xi’an Jiaotong University, Xi’an, China; ^3^ Zonglian College, Xi’an Jiaotong University, Xi’an, China; ^4^ Qide College, Xi’an Jiaotong University, Xi’an, China

**Keywords:** magnetosurgery/magnetic surgery, magnetic anchor technique, laparoscopic cholecystectomy, transumbilical single-port laparoscopy, retrospective study

## Abstract

**Background and objectives:**

Magnetic anchor technique (MAT) is frequently used in laparoscopic cholecystectomy. However, there are few reports on its clinical application in China. In this study, we retrospectively analyzed the clinical application of MAT in laparoscopic cholecystectomy in China.

**Materials and methods:**

25 patients (4 males, 21 females) who underwent laparoscopic cholecystectomy assisted by MAT at the First Affiliated Hospital of Xi'an Jiaotong University were enrolled from November 2020 to March 2021. Their records were retrospectively analyzed. The magnetic anchor device was independently designed and developed by the authors and consisted of the anchor magnet and magnetic grasping apparatus. Surgical time, intraoperative blood loss, intraoperative accidents, operator experience, postoperative incision pain score, postoperative complications, and other indicators were evaluated and analyzed.

**Results:**

All patients successfully underwent laparoscopic cholecystectomy, including 3 cases of MAT-assisted transumbilical single-port LC, 16 cases of MAT-assisted 2-port LC and 6 cases of conventional 3-port LC. The median operation time was 50 min (range 30–95 min); intraoperative bleeding was less than 30 ml. The median score of surgical incision on day 1 and 3 after the operation was 3 (range 1–4) and 1 (range 1–3), respectively. All patients had no intraoperative bile duct injury, vascular injury, postoperative bleeding, bile leakage, biliary stricture and other complications. No adverse events (such as injury to adjacent organs or failure of the magnetic anchor device) occurred either during or after the operation.

**Conclusions:**

The MAT-assisted laparoscopic cholecystectomy appears to be safe, feasible and effective and exhibits unique assistance in transumbilical single-port laparoscopic cholecystectomy.

## Introduction

Benign gallbladder diseases including gallbladder stones, cholecystitis, gallbladder polyposis, and gallbladder adenomyosis are common ailments. Cholecystectomy has remained the treatment of choice for the management of benign gallbladder diseases for more than 100 years. However, in the last three decades, laparoscopic cholecystectomy (LC) has replaced open cholecystectomy and small incision cholecystectomy as the preferred surgical approach for benign gallbladder diseases wherever resources and expertise are available (1, 2). With the improved technology and experience in LC, abdominal wall ports have gradually reduced from the original 4-port system to the more common 3-port system, and more recently, to a single-port LC in some hospitals (3, 4). However, with the reduction of the number of ports in the abdominal wall, many problems have arisen such as the effective exposure of the surgical field and an increase in the interference between operating instruments in the surgical operation (5). Achieving effective tissue retraction and operative field exposure under the reduced port system and improving the ease of operation remains a major issue to be resolved. In addition to improving operating quality, advanced laparoscopic surgical instruments could be crucial for solving this problem.

In modern times, the application of magnetic devices in surgery has been reported increasingly. Among these innovative magnetic devices, many have shown great superiority and subversiveness (6). For example, a specially designed magnetic device can realize vascular anastomosis (7–10), digestive tract anastomosis (11–15), animal model preparation of cystostomy and tracheoesophageal fistula (16, 17). A magnetic device designed based on magnetic anchor technique (MAT) is being used to assist with thoracoscopy and laparoscopic surgery (18, 19), reducing surgical stamping, optimizing surgical operations, and having important clinical application value.

To date, there has been no retrospective clinical study on the use of magnetic anchor devices in laparoscopic cholecystectomy in China. Herein, we report our experiences on the clinical feasibility of a magnetic anchor device in a laparoscopic cholecystectomy.

## Material and methods

### Study protocol and patients

Clinical data from 25 patients who were diagnosed with gallbladder disease and underwent laparoscopic cholecystectomy assisted by MAT at the First Affiliated Hospital of Xi'an Jiaotong University from November 2020 to March 2021 were collected. This study was approved by the Ethics Committee of the First Affiliated Hospital of Xi'an Jiaotong University (No. 2018-W18) and all research was performed in accordance with relevant guidelines/regulations. All patients or their authorized legal representatives gave informed consent for the use of MAT in their operations.

### Patient selection

Inclusion criteria were as follows: (1) diagnosis confirmed by a computerized tomography scan, magnetic resonance imaging of the upper abdomen or B-scan ultrasonography; and (2) informed consent signed by the patient or their family members. The exclusion criteria were as follows: (1) patients with an implanted cardiac pacemaker; (2) patients unable to tolerate general anesthesia during surgery due to severe cardiopulmonary diseases and/or patients with pneumoperitoneum; and (3) patients who were considered inappropriate by the investigators for other reasons.

### Magnetic anchor device

**Figure 1:**
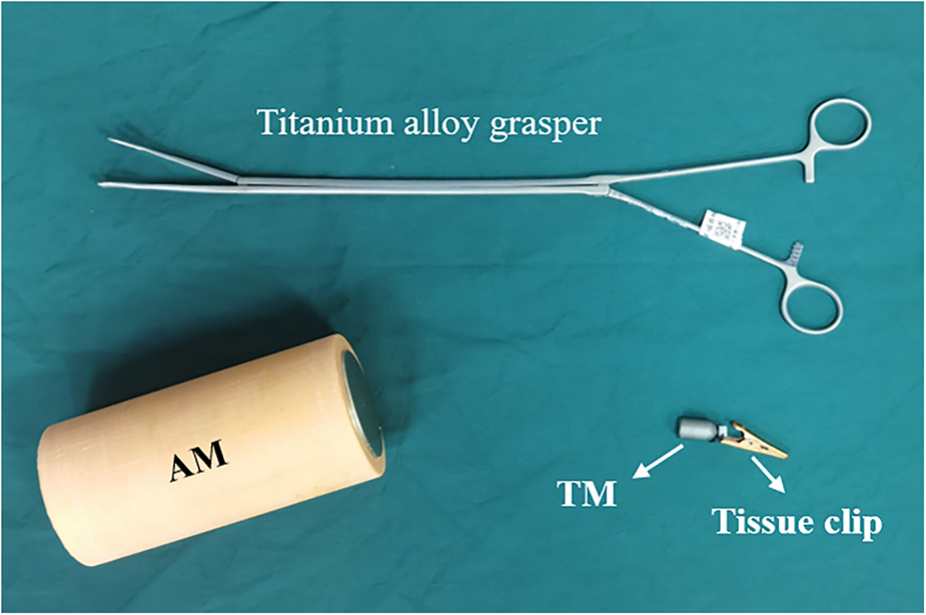
Magnetic anchor device.

The magnetic anchor device was designed and developed by the authors and consists of an anchor magnet (AM) and a magnetic grasping apparatus. The auxiliary operation instrument is a titanium alloy tissue grasping forceps. The AM is a cylindrical magnet with a diameter of 60 mm, a height of 160 mm, and made of N50 sintered-type neodymium-iron-boron. The magnetic grasping apparatus possesses a tissue clip connecting a target magnet (TM) with a silk thread for a total length of 55 mm. The magnetic anchor device is shown in Figure 1. The maximum magnetic force can reach as high as 59.17 N when the AM and the TM are attracted together at zero distance.

### Description of the surgical procedure

Under general anesthesia, all the patients were placed in the supine position. Routine sterilization and drape were performed, and a 10 mm Trocar was formed under the umbilicus to establish pneumoperitoneum. Preliminary predictions were made according to the adhesion around the gallbladder and the degree of gallbladder inflammation. For patients with mild gallbladder inflammation, no obvious adhesion, and good gallbladder triangle exposure, magnetic anchoring single-hole LC was used; otherwise, magnetic anchoring two-port LC was used.

MAT-assisted transumbilical single-port LC: A 2 cm arc incision around the umbilicus was made to enter into the abdomen layer by layer. The single port (Hangzhou Kangji Medical Instrument Co., Ltd.) was then inserted to establish a pneumoperitoneum. The internal grasper was placed through the single-port and clamped the ampulla of the gallbladder. An AM was placed outside the right abdominal wall of the patient and then attracted to the internal grasper and pulled the gallbladder to expose the gallbladder triangle. The hook cautery and small dissector were carefully dissected to separate the cystic duct and cystic artery, which then clipped and disconnected. The position of the AM was adjusted to maintain good gallbladder bed tension. The electric hook was run anterograde along the gallbladder bed to strip the gallbladder, and then the AM was removed. After checking the gallbladder bed for bleeding and bile leakage, the abdominal gas was removed, the single-port port was pulled out, and the incision was sutured.

MAT-assisted 2-port LC: A 12 mm port on the right below the xiphoid process was created to establish pneumoperitoneum, and the internal grasper was placed through the port. The internal grasper clamped the ampulla of the gallbladder, and an AM was placed outside the right abdominal wall of the patient. The AM was then attracted to the internal grasper and pulled the gallbladder to expose the gallbladder triangle. The rest of the surgical procedure was the same as the conventional 3-port LC, except the 5-mm port in the lower edge of right costal arch was omitted, and the magnetic anchor device was used rather than the spring grasping forceps.

Conventional 3-port LC: A 10 mm port under the umbilicus was used to insert the laparoscopic light source and camera system. A 5 mm port in the lower edge of the right costal arch was used to insert the spring grasping forceps. A 10 mm port under the xiphoid process was used to insert the main operating instruments and to remove the gallbladder.

### Statistical analysis

Study parameter indicators included operating time, intraoperative blood loss, damage to the surrounding organ, failure of the magnetic anchoring device, the experience of the operator and assistants, postoperative incision pain score, perioperative complications, and other indicators. SPSS 20.0 software was used for statistical analysis. Normally distributed data were expressed as the mean ± standard deviation, while skewed data were expressed as the median.

## Results

**Figure 2:**
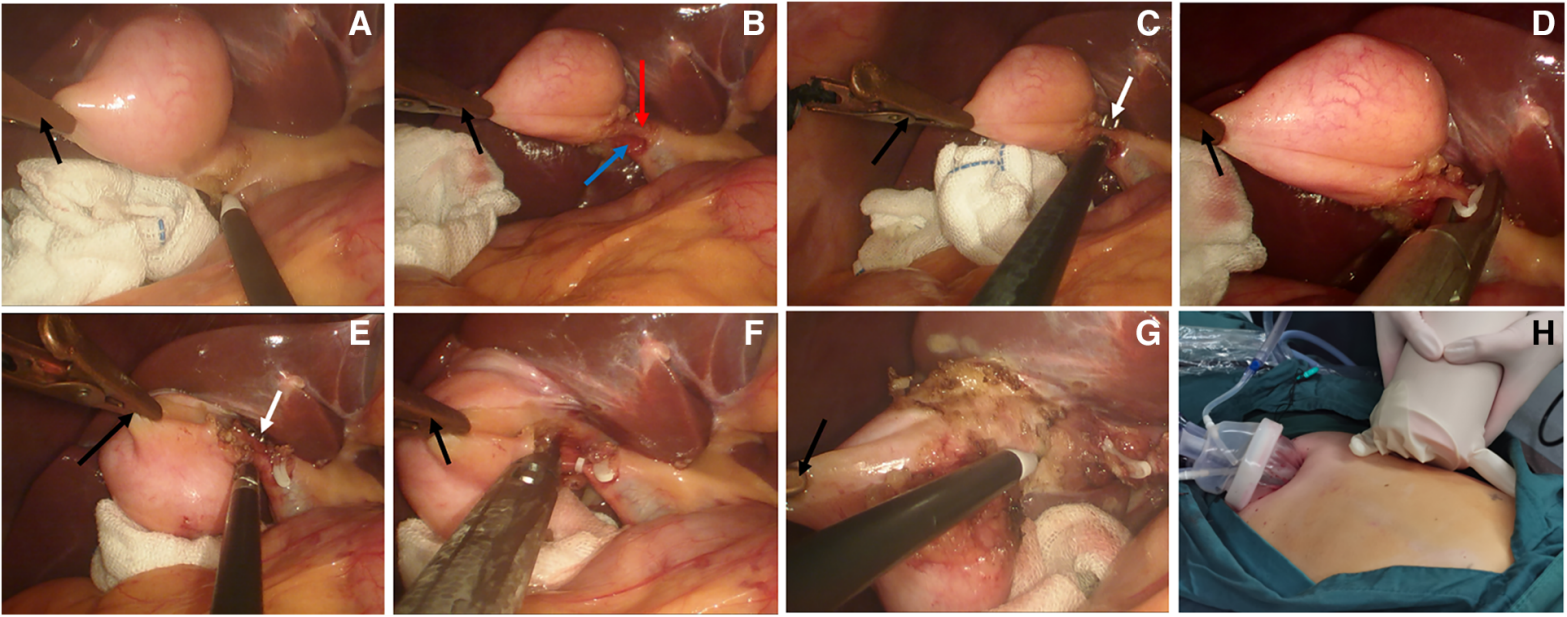
MAT-assisted transumbilical single-port LC operation procedure. (**A**) The gallbladder is pulled by magnetic anchor device, and the trigone structure of the gallbladder was dissected using an electric hook. (**B**) The cystic duct and artery were dissected out. (**C**) The cystic artery was dissected (white arrow). (**D**) The cystic artery was clipped with a vascular clip. (**E**) The cystic duct was dissected (white arrow). (**F**) Clamping of the cystic duct. (**G**) Anterograde dissection of the gallbladder bed. (**H**) The single port and anchor magnet located in the patient's upper abdomen (the black arrow in the figure is the magnetic anchors built-in grasping forceps, the red arrow is the cystic artery, and the blue arrow is the cystic duct).

**Figure 3:**
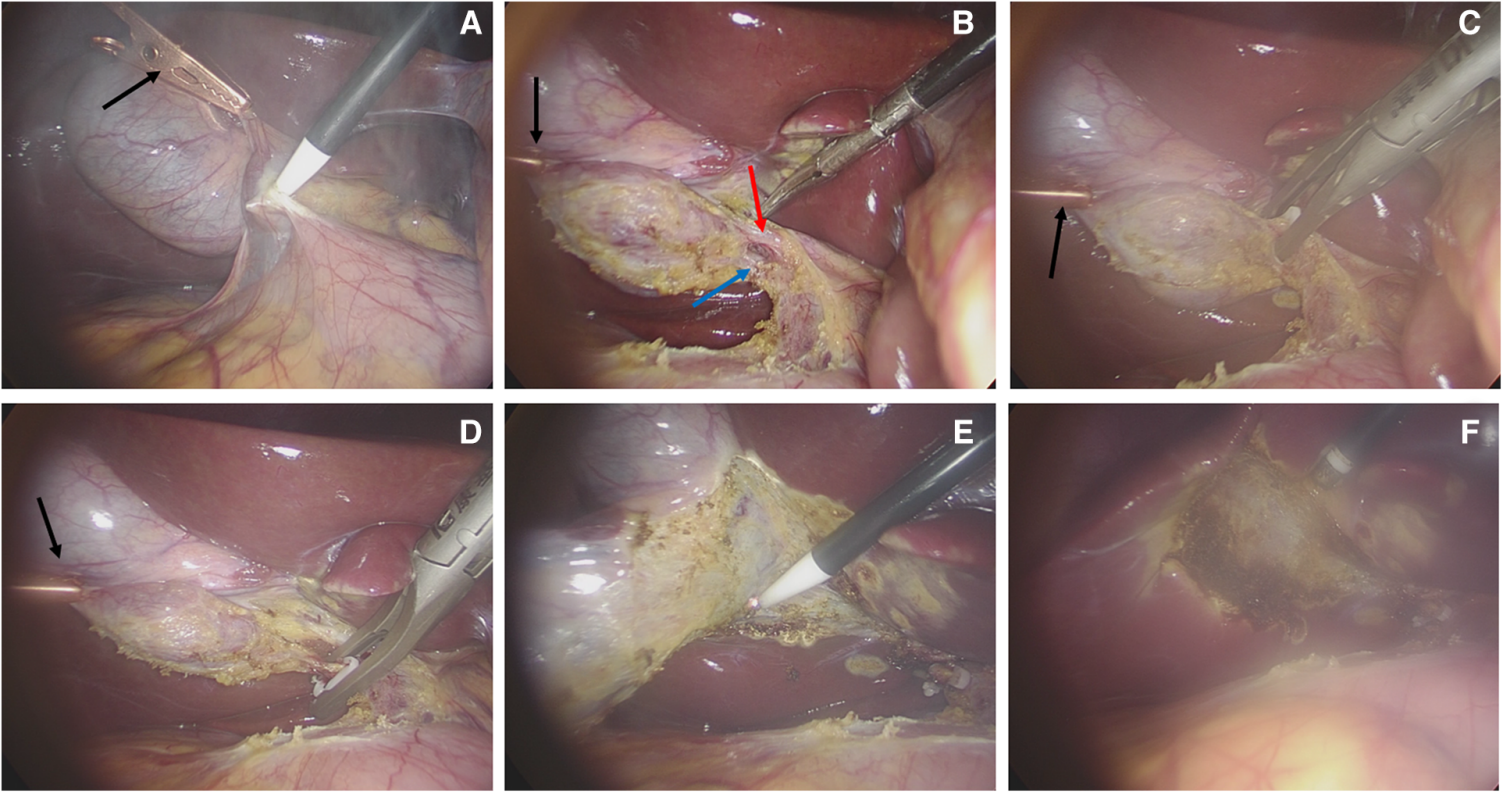
MAT-assisted 2-port LC operation procedure. (**A**) The gallbladder was pulled by the magnetic anchor device, and the adhesion around the gallbladder was separated by the hook cautery. (**B**) The cystic artery and the cystic duct were dissected. (**C**) The cystic artery was clamped by the vascular clip. (**D**) The cystic duct was clipped by the vascular clip. (**E**) Stripping the gallbladder bed. (**F**) The gallbladder bed after cholecystectomy (the black arrow in the figure is the magnetic anchors built-in grasping forceps, the red arrow is the cystic artery, and the blue arrow is the cystic duct).

A total of 25 patients (4 males, 21 females) with a median age of 37 years (range 25–71 years); BMI 25.24 ± 3.77 kg/m^2^ (range 19.57–33.30 kg/m^2^) were included in the study. Ten patients had cholecystolithiasis with chronic cholecystitis, 3 had gallbladder polyps, 1 had gallbladder adenomyosis, and 11 patients had cholecystolithiasis with an acute attack of chronic cholecystitis. Three of the 25 patients underwent MAT-assisted transumbilical single-port LC, 16 patients underwent MAT-assisted 2-port LC, and 6 patients underwent conventional 3-port LC. None of the patients underwent open cholecystectomy. The median operating time was 50 min (range 30–95 min): the 3 patients with MAT-assisted transumbilical single-port LC had a median operating time of 95 min (range 40–95 min), the 16 patients with MAT-assisted 2-port LC had a median operating time of 50 min (range 30–90 min), and the 6 patients with conventional 3-port LC had a median operating time of 70 min (range 40–90 min). Intraoperative blood loss was less than 30 ml. There was no injury in the blood vessels, bile duct or intestine reported during the operation. Figures 2, 3 show the MAT-assisted transumbilical single-port LC and MAT-assisted 2-port LC procedures.

The median score of surgical incision pain (based on the Wong-Banker facial scale method) in all the patients was 3 (range 1–4) and 1 (range 1–3) on day 1 and day 3 after surgery, respectively. Among them, 3 patients with MAT-assisted transumbilical single-port LC had moderate surgical incision pain. The median score was 1 (range 1–2) and 1 (range 1–1) on postoperative days 1 and 3, respectively. The median score of incision pain in 16 patients with MAT-assisted 2-port LC was 3 (range 2–4) and 1 (range 1–2) on day 1 and day 3 after surgery, respectively. The median pain score of surgical incision in 6 patients with conventional 3-port LC in transition on day 1 and day 3 after surgery was 3.5 (range 2–4) and 2 (range 1–3), respectively. 14 patients had a surgical incision pain score ≥ 3, 10 patients reported that the main site of pain was located under the xiphoid process, 4 patients reported that the main site of pain was under the right costal margin, and 0 patients reported the main site of pain as the umbilicus.

During the perioperative and 6-month follow-up period, there were no postoperative complications reported in any of the patients, including bile leakage, biliary stricture, wound infection, or port-site hernia.

## Discussion

Minimally invasive surgery is an important trend in the advancement of modern surgery. It is the goal of the surgeon to ensure the safety of patients by reducing trauma as much as possible. In laparoscopic surgery, further reduction in the number of abdominal wall ports not only reduces postoperative pain in patients, but also has cosmetic advantages by reducing abdominal scarring. The reduction of abdominal wall ports means an increase in the difficulty of operation, among which the chopstick effect is the most important influencing factor. Reducing the number of laparoscopic instruments entering is the key to overcoming the chopstick effect. Some researchers have used the abdominal wall hanging wire method and special devices to traction the organs (20–22), which can optimize the operation, however, the effect has been limited.

Magnetic anchor technique (MAT) utilizes the magnetic field attraction between two magnets, or between magnets and paramagnetic substances, so that the anchor magnet can anchor to the target magnet without contact. The main function of the magnetic anchoring device in laparoscopic surgery is to replace the spring grasping forceps, so as to reduce the number of ports. Magnetic anchoring devices have been reported and applied abroad. The novel magnetic anchoring device used in this study was independently designed and developed by the authors.

This study shows that the use of magnetic anchor device can successfully complete the port-reduced LC without accidental injury and postoperative complications. In this study, only 3/25 patients underwent MAT single-port LC, which accounts for a small proportion. The main reason was that it depended on the patients' preoperative treatment demands. They were also influenced by the inflammation of the gallbladder observed during the operation. A single-port is required for single-port LC, but it costs more than conventional trocars, leading some patients to choose two-port LC. Among the 6 patients with the conventional three-port LC, 4 patients were converted because the gallbladder was in the acute inflammation stage, the gallbladder wall was edematous and thickened, and the gallbladder tension was large and the magnetic anchor device was unable to maintain effective tissue tension. The remaining two patients had variations in the anatomical structure of the gallbladder triangle and it was difficult to accurately and effectively identify Calot's triangle using the MAT 2-port LC, thus the conventional three-port method was used. The remaining 16 patients successfully underwent MAT 2-port LC. Due to the small number of cases in this group, the surgeon had not yet crossed the learning curve, so the operation time was slightly longer. However, with experience and time it is expected that there will be a reduction in overall procedure time.

This study showed that the incision pain scores of patients with MAT-assisted transumbilical single-port LC and MAT-assisted 2-port LC were lower than those of conventional 3-port LC patients at day 1 and 3 after surgery. In patients with an incision pain score greater than or equal to 3, the main pain sites were the right subcostal and subxiphoid. The magnetic anchor technique in LC avoids the right subcostal and the subxiphoid. This has great significance for prompting patients to recover quickly and return to normal activity as soon as possible after the surgery.

In this study, 6 patients were transferred to conventional 3-port LC which is a rate higher than that of other previously reported studies (19, 23). The main reason for that could be the inclusion criteria for patients in this study were set lower. The main purpose of this was to maximize the application potential of the magnetic anchor technique in LC. The results show that the magnetic anchor technique is not suitable for acute cholecystitis complicated with gangrenous perforation and severe intra-abdominal adhesions. However, this study has several limitations including: single center, small sample size, and the retrospective nature of the study. We believe that this technique has a promising future. With more experience gained in the clinical application of MAT-assisted LC, the complication rate and rate of transfer to conventional LC are expected to reduce. Further studies, especially randomized controlled clinical trials are necessary before final recommendations are made.

## Conclusion

In conclusion, this study showed that the MAT is safe, feasible and effective for LC. The development of appropriate patient inclusion criteria has important guiding significance for the clinical development of magnetic anchor technique-assisted LC.

## Data Availability

The raw data supporting the conclusions of this article will be made available by the authors, without undue reservation.
